# Revisiting the female germline cell development

**DOI:** 10.3389/fpls.2024.1525729

**Published:** 2025-01-14

**Authors:** Youmei Huang, Yunlong Zhang, Jiahong Yang, Xinpeng Xi, Yanfen Liu, Hanyang Cai, Yuan Qin

**Affiliations:** College of Life Sciences, Fujian Provincial Key Laboratory of Haixia Applied Plant Systems Biology, State Key Laboratory of Ecological Pest Control for Fujian and Taiwan Crops, Haixia Institute of Science and Technology, Fujian Agriculture and Forestry University, Fuzhou, China

**Keywords:** MMC, plant reproduction processes, epigenetic pathways, cell-cycle regulators, phytohormones

## Abstract

The formation of the female germline is the fundamental process in most flowering plants’ sexual reproduction. In *Arabidopsis*, only one somatic cell obtains the female germline fate, and this process is regulated by different pathways. Megaspore mother cell (MMC) is the first female germline, and understanding MMC development is essential for comprehending the complex mechanisms of plant reproduction processes. Recently, more advanced technologies such as whole-mount single-molecule fluorescence *in situ* hybridization (smFISH), laser-assisted microdissection (LCM), chromatin immunoprecipitation/sequencing, and CRISPR gene editing have provided opportunities to reveal the mechanism of female germline development at different stages. Single-cell transcriptome/spatial transcriptomics analysis helps to investigate complex cellular systems at the single-cell level, reflecting the biological complexity of different cell types. In this review, we highlight recent progress that facilitates the development of the female germline to explore the roles of crucial gene regulatory networks, epigenetic pathways, cell-cycle regulators, and phytohormones in this process. This review discusses three key phases in female germline development and provides the possibility of distinct pathways restricting germline development in the future.

## Introduction

**Figure 1:**
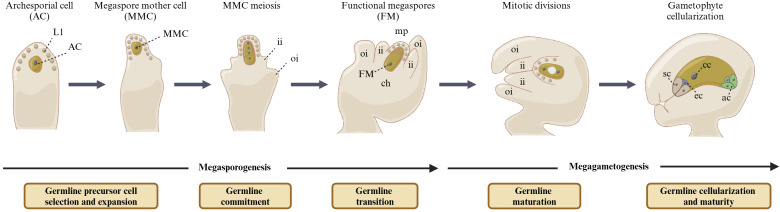
Female gametophyte development in *Arabidopsis thaliana*. The female germline took place in the distal domain of ovule. Only one subepidermal cell elongated and expanded to form archesporial cells (ACs) and further specialized into megaspore mother cells (MMCs). The MMCs underwent one meiotic division to form four haploid megaspores. Among them, three megaspores near the micropore end experienced degeneration rapidly, while only the megaspores at the chalazal end survived and successfully developed into functional megaspores (FMs). This stage in female germline development is referred to as megasporogenesis. The FMs underwent three rounds of continuous mitosis, which led to the production of a mature female gametophyte (FG), which contains four different cell types, including three antipodal cells, one central cell, two synergid cells, and one egg cell. This stage in female germline development is referred to as megagametogenesis. ii, inner integument; oi, outer integument; ch, chalaza; mp, micropyle; ac, antipodal cells; cc, central cell; sc, synergid cells; ec, egg cell.

The flowering plants exhibit a complex life cycle that alternates between diploid (sporophytic/somatic) and haploid (gametophytic) generations (Pinto et al., 2019). The reproductive cells of plants are usually re-evolved from somatic cells in the reproductive organs of flowers, such as pistils and stamens. In *Arabidopsis*, the formation of the female germline generally begins with the differentiation of a subepidermal cell at the top of the ovule primordia, which elongates and expands to form archesporial cell (AC) and further specialize into megaspore mother cell (MMC). The MMC undergoes one meiotic division to form four haploid megaspores. Among them, three megaspores near the micropore end experience programmed cell death, while only the megaspores at the chalazal end survive and successfully develop into functional megaspores (FMs). This stage in female germline development is referred to as megasporogenesis. Subsequently, FMs undergo the stage of megagametogenesis, which involves three rounds of continuous mitosis and leads to the production of a mature female gametophyte (FG), also known as the megagametophyte or embryo sac. The mature FG contains four different cell types, including three antipodal cells, one central cell, two synergid cells, and one egg cell. Both the egg cell and the central cell are fertilized, producing an embryo and an endosperm, respectively (**Figure 1**) (Yan et al., 2014; Cai et al., 2022b).

**Figure 2:**
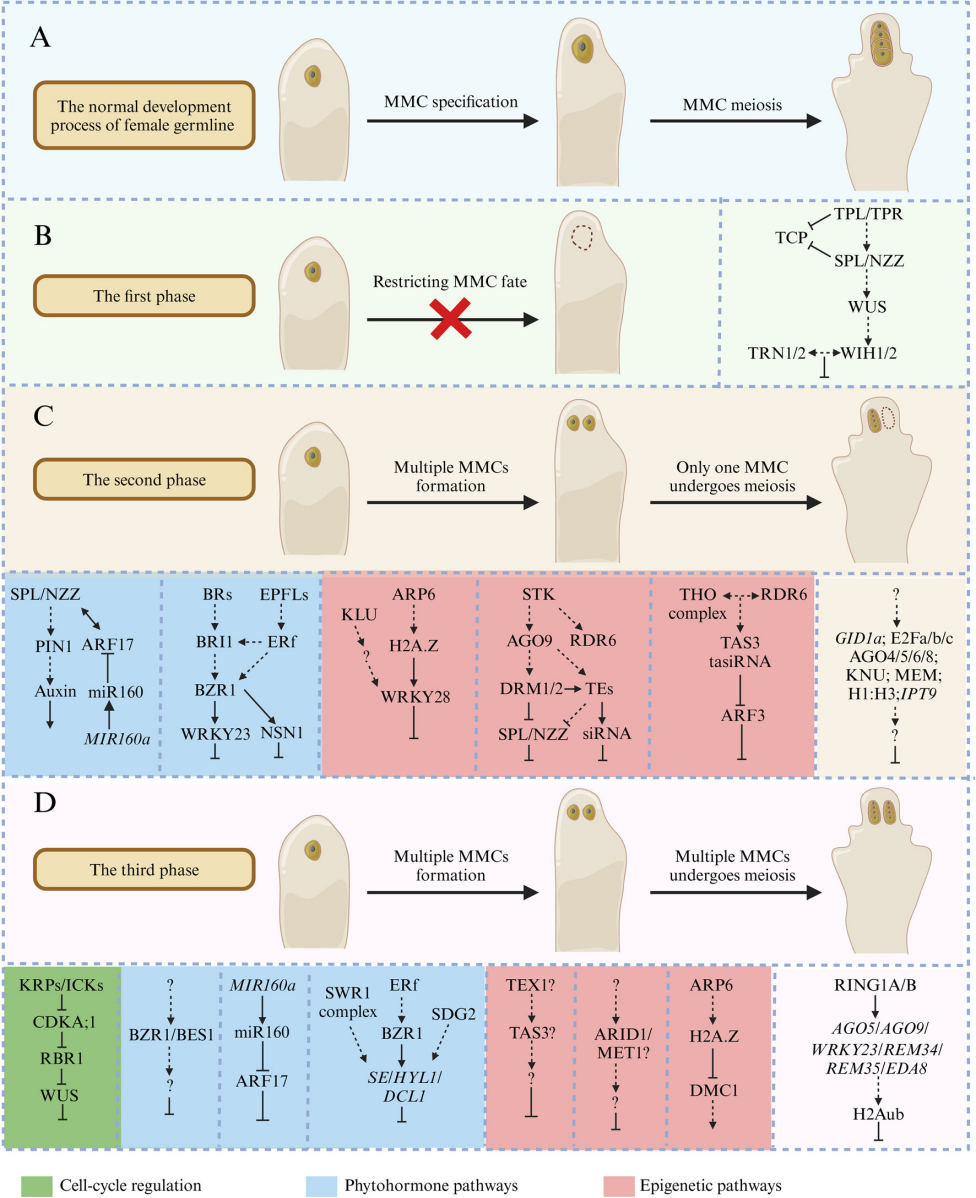
The key factors and mechanisms that regulate megaspore mother cell (MMC) development in *Arabidopsis*. **(A)** The normal development processes of female germline in *Arabidopsis*. **(B)** The first phase is related to the initiation of the female germline and regulates cell expansion. The absence of key genes can prevent female germline formation in ovule primordia, such as SPL-WUS-WIH1/2 pathway. **(C)** The second phase restricts the female germline into a single MMC in ovule primordia. The related mutants will form multiple MMCs in ovule primordia, but only one MMC can undergo meiotic division, such as KLU-ARP6-WRKY28 signaling module and BR/BRI1-EPFL/ERf-BZR1 pathway. **(D)** The third phase controls the mechanisms of MMC entry meiotic divisions and the subsequent three rounds of mitotic divisions, such as KRP/ICK-CKA;1-RBR1-WUS pathway. The green box represents cell-cycle regulation, the blue box represents plant hormone pathways, and the red box represents epigenetic pathways.

The development of female gametophytes is a crucial step in the sexual reproduction process of most flowering plants. In *Arabidopsis*, only one somatic cell can obtain the female germline fate, and the program of somatic cells’ perception and response to germline-inducing signals is strictly controlled. Based on the phenotype analysis of ovule mutants, the progress of MMC development can be divided into three phases (or checkpoints). The first phase is related to the initiation of the female germline and regulates cell expansion. The second phase restricts the female germline into a single MMC in the ovule primordia, and the third phase controls the mechanisms of MMC entry into meiotic divisions and the subsequent three rounds of mitotic divisions (Pinto et al., 2019). In this review, we highlight the recent achievements to understand the mechanism of female germline development based on the aspects of i) the establishment of female germline identity, ii) ectopic acquisition of MMC identity, and/or iii) continued ectopic germline development (**Figure 2**).

## The key factors function in the initiation of female germline identity

There are several essential genes in the establishment of the female germline, and the absence of these genes can prevent female germline formation. In *Arabidopsis*, *SPOROCYTELESS/NOZZLE* (*SPL/NZZ*) has been early demonstrated to play an important role in the process of somatic to germline transformation, and *spl/nzz* mutants can form archesporial cells in both anther and the ovule primordia, but these cells fail to differentiate into pollen mother cells (PMCs) and MMCs (Schiefthaler et al., 1999; Yang et al., 1999). *SPL/NZZ* encodes a nuclear localization protein homologous to the MADS box transcription factors and a putative MYC-type helix–loop–helix dimerization domain signature at the carboxy-terminal (Yang et al., 1999). A further study has found that SPL/NZZ functions as an adaptor-like transcriptional repressor using its EAR motif at the C-terminal end to recruit TOPLESS/TOPLESS-RELATED (TPL/TPR) corepressors to inhibit the activities of CINCINNATA (CIN)-like TEOSINTE BRANCHED1/CYCLOIDEA/PCF (TCP) transcription factors during MMC formation (**Figure 2B**) (Chen et al., 2014; Wei et al., 2015). Conversely, loss of TPL1 function and overexpression of TCP transcription factors result in no MMC formation in the ovule primordia, which is similar to the phenotype of the *spl/nzz* mutant (**Figure 2B**) (Wei et al., 2015).

WUSCHEL (WUS) transcription factor is best known for its function to maintain stem cell fate in the shoot apical meristem (SAM) (Mayer et al., 1998), which also plays a key role in MMC formation. Like *spl/nzz* mutants, *wus* mutants also lack a primary germline cell in the ovule primordia. Furthermore, both *WUS* and *SPL/NZZ* are expressed in the apical epidermal cell layer before MMC initiation (**Figure 2B**), indicating that *SPL/NZZ* and *WUS* may function in the same mechanism in MMC development and establish an environment for germline formation through cell autonomous components (Yang et al., 1999; Gross-Hardt et al., 2002; Lieber et al., 2011). *SPL/NZZ* was determined to act upstream of *WUS* since the expression of *WUS* is reduced in *spl/nzz* mutants (Yang et al., 1999; Lieber et al., 2011). Further research has found that *WUS* can regulate the expression of two redundantly acting genes, *WINDHOSE 1* (*WIH1*) and *WIH2*, and the simultaneous absence of these two genes leads to loss of the MMC in the ovule primordia. *WIH1* and *WIH2* encode small peptides that may function as ligands for the tetraspanin-type transmembrane protein TORNADO 2 (TRN2)/EKEKO and the leucine-rich repeat (LRR) protein TRN1/LOPPED 1 in promoting MMC formation (**Figure 2B**) (Lieber et al., 2011). However, the interaction between WIH and TRN in promoting the transition of somatic to female germline still needs to be demonstrated.

The number of ACs or MMCs is strictly limited during the development of ovules. In other flowering plants, they appear to use a lateral inhibition mechanism that allows the MMC to repress germline cell fate in its surrounding cells, thereby regulating the number of MMC. In rice, *TAPETUM DETERMINANT-LIKE 1A* (*OsTDL1A*) encodes a class of small peptides that are preferentially expressed in MMC (Zhao et al., 2008). *MULTIPLE SPOROCYTE* (*OsMSP1*) encodes a leucine-rich-repeat receptor kinase, expressed in the L1 layer cells surrounding MMC (Nonomura et al., 2003). Further research has found that OsTDL1A can directly bind to OsMSP1, inhibiting the transformation of somatic cells around MMC into female germline (Zhao et al., 2008). In maize, *MULTIPEARCESPORIAL CELLS 1* (*MAC1*), the *OsTDL1A* homologous gene, plays roles in the switch of the hypodermal cells from the vegetative to the meiotic (sporogenous) pathway in ovule development (Sheridan et al., 1996; Wang et al., 2012). In the *mac1* mutant, several hypodermal cells develop into archesporial cells, and the resulting megasporocytes undergo normal meiosis, ultimately developing into embryo sacs (Wang et al., 2012).

## Epigenetic pathways involved in female gametophyte development

Epigenetic reprogramming is widely present in female gametophyte development. ARGONAUT (AGO) is an essential component of the RNA-directed DNA methylation (RdDM) mechanism, which can regulate mRNAs during miRNA- or siRNA-guided post-transcriptional gene silencing (Havecker et al., 2010; Olmedo-Monfil et al., 2010). AGO9 interacts with 24-nt small RNAs (sRNAs) to silence transposable elements (TEs) in the nucellus to control the specification of germline cells (Olmedo-Monfil et al., 2010). In a previous study, AGO9 had abundant expression in the epidermal cell layer (L1), and further study found that the nucleus of the MMC in several ecotypes sporadically shows AGO9 expression, suggesting that a transient nuclear AGO9 localization can be found in the MMC (Rodriguez-Leal et al., 2015). Furthermore, the expression patterns of AGO9 localized in the multiple abnormal gamete precursors of *rdr6* mutants share a cellular identity with the gamete precursors found in selected ecotypes. These results indicate that AGO9 is involved in epigenetic silencing that controls the specification of female gamete precursors, which may be used to indicate the formation of germ cells (Rodriguez-Leal et al., 2015). Furthermore, approximately 50% of *ago9* mutant ovules display supernumerary MMCs (**Figure 2C**). Therefore, it is speculated that AGO9 may bind to the 24-nt sRNAs to regulate TEs in MMC and its accessory cells, but different cell types may affect sRNA accumulation or gene silencing partners, which may be the reason for the production of multiple MMCs in the *ago9* mutant (Olmedo-Monfil et al., 2010). Further studies found that loss-of-function mutants of other AGO proteins, including AGO4, AGO6, and AGO8, also exhibit multiple MMC-like cells in the ovule primordia, but the identity of these cells still needs to be further determined. These results support the role of the RNA-based silencing mechanism in preventing the abnormal specification of multiple premeiotic gametophytic precursors during early ovule development (**Figure 2C**) (Hernandez-Lagana et al., 2016). A recent report found that the *Arabidopsis* mutant alleles *mir822-1* and *mir822-2* display extra FMs and divide without giving rise to differentiated female gametophytes (Tovar-Aguilar et al., 2024). Overexpression of *miR822* target genes *At5g02350*, *At5g02330*, and *At2g13900* show similar defects equivalent to those found in *mir822* mutant plants, and these three microRNA822 (miR822) target genes are overexpressed in *ago9* mutant ovules, indicating that *miR822* acts through an AGO9-dependent pathway to modulate monosporic development in *Arabidopsis* (Tovar-Aguilar et al., 2024).

RNA DEPENDENT POLYMERASE 6 (RDR6) and SUPPRESSOR OF GENE SILENCING 3 (SGS3) are essential parts of the biogenesis of *trans*-acting small interfering RNAs (tasiRNAs) (Yoshikawa et al., 2005). The mutation of *RDR6* and *SGS3* also showed an identical phenotype to *ago9* mutants with supernumerary MMCs in the ovule primordia, which suggests that the movement of sRNA silencing out of somatic companion cells is necessary for the specification of the MMC (**Figure 2C**) (Olmedo-Monfil et al., 2010). These results suggest that AGO9 controls gametic cell commitment by acting in a non-cell autonomous sRNA-dependent pathway in ovule development (Yoshikawa et al., 2005; Olmedo-Monfil et al., 2010). In maize, AGO4 is likely a functional homolog of *Arabidopsis* AGO9, which is necessary for non-CG DNA methylation at centromeric and knob heterochromatin (Singh et al., 2011). AGO104 accumulates specifically in somatic cells surrounding the female meiocyte. The absence of AGO104 gives rise to unreduced gametes but not a multiple MMC phenotype. A further study found that the unreduced gametes underwent a mitotic rather than a meiotic division, consequently developing unreduced gametes (Singh et al., 2011). Another gene that plays a similar role is *MNEME* (*MEM*), which encodes for a putative ATP-dependent RNA helicase of the DEAD-box family that is also involved in the establishment of the epigenetic landscape in the female gametophyte (Schmidt et al., 2011). *MEM* is specifically expressed in MMC, and the mutation of *MEM* results in the formation of multiple MMC-like cells in the ovule primordia. However, whether the enlarged MMC-like cells in the *mem* mutant acquire MMC identities still needs to be further determined. Moreover, several *mem* mutants exhibit altered epigenetic modifications in gametophytic nuclei (**Figure 2C**). These results suggest that the potential function of MEM is in inhibiting germline fate in somatic cells and establishing a germline-specific chromatin state, but the mechanism is still unclear (Schmidt et al., 2011).

It is known that the RdDM pathway can regulate gene expression via AGO-mediated mRNA degradation or cytosine DNA methylation of target genes by DOMAINS REARRANGED METHLTRANSFERASES1 (DRM1) and DRM2 methylases (Marston, 2014). The *pKNU::nlsYFP* transcriptional marker specifically marks cells possessing MMC identity. *drm1 drm2* double mutants also present supernumerary MMC-like cells in the ovule primordia, but only one cell expressed *pKNU::nlsYFP* signal, which is similar to other mutants of the RdDM pathway (Marta et al., 2020). *SPL/NZZ* is ectopically expressed in *ago9*, *rdr6*, and *drm1drm2* mutants, and *SPL/NZZ* is essential for MMC differentiation, indicating that the excessive MMC-like cell development may be due to the ectopic activation of *SPL/NZZ* (**Figure 2C**) (Marta et al., 2020). Recently, the RdDM pathway has been reported connected to SEEDSTICK (STK) to control ovule development. SEEDSTICK (STK) is a MADS-box transcription factor, which is expressed in many sporophytic cell types, including the nucellus, chalaza, and integuments (Matias-Hernandez et al., 2010). In *stk* mutants, nearly one-half of ovules contain multiple MMC-like cells, but only one cell expressed *pKNU::nlsYFP* signal. A further study found that *STK* directly regulates the expression of *AGO9* and *RDR6* in the ovule and therefore indirectly *SPL/NZZ* expression (**Figure 2C**) (Marta et al., 2020). Although *stk*, *ago9*, *rdr6*, and *drm1 drm2* mutants display excessive MMC-like cells in the ovule primordia, only one MMC can enter the meiotic program (Marta et al., 2020).

In most sexual flowering plants, the female gametogenesis is initiated from the subepidermal L2 somatic cell that undergoes a fate transition from somatic cells to germ cells (Yang and Sundaresan, 2000). This process is accompanied by large-scale chromatin reprogramming, such as chromatin decondensation, heterochromatin reduction, depletion of linker histones, and core histone variants change, which may establish an epigenetic and transcriptional status distinct from surrounding somatic cells (She et al., 2013). There are 15 H3-related genes of *HISTONE THREE RELATED* (*HTR*) in *Arabidopsis* (Okada et al., 2005). *HTR13* typically encodes an H3.1 variant associated with inactive transcription, and most H3.1 histones are incorporated into the chromatin of proliferating tissues during the S-phase of DNA replication. Furthermore, H3.1 eviction is considered a characteristic of cell-cycle exit event that leads to pluripotent stem cell fate or cell differentiation. In the early stage of ovule development, H3.1 can be evicted in multiple subepidermal cells that may develop into MMC, and this state persists only in the MMC (Jacob et al., 2014). Therefore, the dynamic expression of H3.1 in the ovule primordium distinguishes the female germline from somatic cells and marks cell fate transition from somatic to germ cell (**Figure 2C**) (Jacob et al., 2014; Hernandez-Lagana and Autran, 2020). Polycomb repressive complex 1 (PRC1) usually catalyzes histone H2A monoubiquitination (H2Aub) to repress gene transcription (Yin et al., 2021). In *Arabidopsis*, RING1A and RING1B are the PRC1 complex catalytic subunits, and knockout *RING1A* and *RING1B* together cause severe defects in the formation of MMC and FM and subsequent mitosis of FM (**Figure 2D**) (Lv et al., 2024). The female gametophyte development essential genes, including *AGO5*, *AGO9*, *WRKY23*, *REM34*, *REM35*, and *EDA8*, were ectopically expressed in *ring1a ring1b* double mutants. The H2Aub levels at these loci were reduced in *ring1a ring1b* mutants, indicating that RING1A/B promotes H2Aub at genes that regulate female gametophyte development (**Figure 2D**) (Lv et al., 2024).

ACTIN-RELATED PROTEIN 6 (ARP6) is one of the subunits of ATP-dependent chromatin remodeling complex SWI2/SNF2-RELATED 1 (SWR1) (Wu et al., 2005). The mutation of *ARP6* leads to the defects of female meiosis in *Arabidopsis*, including aberrant centromere pairing, loss of homologous chromosome pairing, and reduction in normal divalents (Rosa et al., 2013; Qin et al., 2014). DMC1 is a key recombinase for efficient pairing of homologous centromeres, which promotes the recombination of the sister chromosomes during meiosis (Da Ines et al., 2012). A further study found that *DMC1* is significantly upregulated in the ovules of *arp6* mutants, and H2A.Z was not enriched at any position of the *DMC1* locus. These results indicate that ARP6 regulates the expression of *DMC1* by modulating the deposition level of H2A.Z at the *DMC1* locus, thereby affecting the meiotic divisions of the female germline (**Figure 2D**) (Qin et al., 2014). The cytochrome P450 gene *KLU* (also known as *KLUH/CYP78A5*) is preferentially expressed in the inner integument. Recent research has found that ARP6 mediates the incorporation of the histone variant H2A.Z at *WRKY28* to promote its expression, and this process is dependent on KLU. *WRKY28* encodes a zinc-finger WRKY TF, which acts downstream of *KLU* and *ARP6* and is significantly reduced in *arp6 klu* double mutants. A further study found that *WRKY28* is exclusively expressed in the hypodermal somatic cells surrounding MMC and inhibits these cells from acquiring MMC identity (**Figure 2C**) (Zhao et al., 2018). SET DOMAIN GROUP 2 (SDG2), the main H3K4 methyltransferase, is involved in various biological processes of plant development (Berr et al., 2010; She et al., 2013). The immunostaining results showed that the activated H3K4me3 histone modification was enriched in MMC (She et al., 2013). A recent report found that SWR1 and SDG2 cooperate with the ERECTA (ER) receptor kinase signaling pathway to control female germline development by restricting the MMC cell fate to a single cell in the ovule primordium (**Figure 2C**) (Cai et al., 2023a).

## Cell-cycle regulators facilitate correct progression of a germline program

Unlike humans and animals, plants do not set aside a specialized germline that produces meiocytes in early embryogenesis. Instead, flowering plants need to specify somatic cells to undergo meiosis. Some cell-cycle regulators control the process of MMC entering meiosis. In the mutants of these cell-cycle regulators, the designated MMC undergoes several mitotic divisions, resulting in the formation of supernumerary MMCs. Cyclin-dependent kinases (CDKs) are the universal drivers of cell-cycle transitions, which promote G1- to S-phase transition and activate genome duplication (De Veylder et al., 2001). The KIP RELATED PROTEINs/INHIBITOR OF CYCLIN-DEPENDENT KINASEs (KRPs/ICKs) are plant CDK inhibitors and modulate CDK enzymatic activity through direct protein binding, and the concentration or level of the ICK/KRP protein is likely important for its function (Wang et al., 1997; De Veylder et al., 2001; Zhou et al., 2003; Juan Antonio et al., 2011). In the cell cycle, the inhibitory effect of Rb homolog RETINOBLASTOMA ASSOCIATED 1 (RBR1) is inhibited by cyclin-dependent kinase A;1 (CDKA;1) when it is phosphorylated. Recent studies have shown that *KRP4*, *KRP6*, and *KRP7* act redundantly in the MMC to repress CDKA;1-dependent inactivation of the RBR1, and RBR1 directly inhibits *WUS* activity and promotes MMC to enter meiosis (**Figure 2D**) (Zhao et al., 2017). In the *rbr1* mutants and *krp4 krp6 krp7* triple mutants, multiple MMCs are formed, and the mitotic reporter CYCB1;2-GFP was observed in excessive MMCs, indicating that the failure of cell division transition from mitosis to meiosis may lead to the formation of excessive MMC (**Figure 2D**) (Zhao et al., 2017). ARID1, an AT-rich interacting domain transcription factor, exhibits a range of epigenetic regulation during cell differentiation (Deborah et al., 2005; Zheng et al., 2014). METHYLTRANSFERASE 1 (MET1) is required to maintain CG methylation, which plays a critical role in silencing transposable elements and regulating gene expression (Liu et al., 2021). It was found that MET1 is inhibited by ARID1 in MMC specification during female gametophyte development, but the mechanism remains unknown (**Figure 2D**) (Li et al., 2017). RBR1 was found to repress MET1 in the female gametophyte (Pauline et al., 2008). Therefore, future studies may consider whether ARID1-mediated MET1 inhibition in the female gametophyte is functionally related to the RB pathway.

Like *rbr1* mutants, the *ick1 ick2 ick3 ick4 ick5 ick6 ick7* septuple mutants (name as *ick1/2/3/4/5/6/7*) display more than one MMC and in the selective survival of FM. The origin of multiple MMCs in *ick1/2/3/4/5/6/7* septuple mutants may result from mitotic division of the MMCs (**Figure 2D**) (Cao et al., 2018). E2F transcription factors have been reported to be involved in the G1–S-phase transition and play important roles in mammalian cell fate determination (Shirley and Doron, 2008). E2Fs are bound to and repressed by RBR1 and thereby block cell proliferation regulatory activity (Desvoyes and Gutierrez, 2020). In *Arabidopsis*, *E2Fa*, *E2Fb*, and *E2Fc* play redundant roles in plant germline formation. The *e2fa e2fb e2fc* triple mutants also form supernumerary MMCs as observed in *rbr1* mutants, which implicates that E2Fs may be necessary to induce its repressor factor RBR1 (**Figure 2C**). In the *e2fa e2fb e2fc* triple mutant, the activity of RBR1 will decrease, leading to the formation of supernumerary MMCs (Yao et al., 2018). However, E2Fs and RBR1 may function in different pathways to regulate MMC specification. In addition, TRIMETHYLGUANOSINE SYNTHASE1 (TGS1) may also be involved in the process of cell division. The *tgs1* mutants contain multiple enlarged cells, but the MMC marker gene *pKNU* was active in only one of them, suggesting that only the cell obtains female fate and enters meiosis (Lorena et al., 2023). Furthermore, the ovules of *tgs1* mutants typically contained tetrads with the two chalaza megaspores specified for gametophytic development, while the micropylar spores will degenerate (Lorena et al., 2023).

## Phytohormones may provide an environment supporting female germline development

Phytohormones are involved in various aspects of plant development, including the development of female germline (Cai et al., 2023b). Auxin signaling output was traced to megasporogenesis, and the auxin reporter DR5:GFP is only observed in one to three apical epidermal cells of the ovule primordium, suggesting a gradient of auxin in apical epidermal cells (Pagnussat et al., 2009). Auxin accumulation in a single cell will promote the expression of auxin response genes mediated by the transcription factor ARF (Auxin RESPONSE FACTOR) (Su et al., 2017). The auxin exporters PIN (PIN-FORMED) play unique roles in response to environmental and developmental signal transportation, resulting in localized changes in auxin concentration and distribution (Justyna et al., 2006). The expression of PIN1 is impaired in *spl/nzz* mutants, indicating that *SPL/NZZ* is involved in maintaining auxin homeostasis (Bencivenga et al., 2012). The recent report found that microRNA160 (miR160) targeted gene *ARF17* (*AUXIN RESPONSE FACTOR17*) genetically interacts with the *SPL/NZZ* function in promoting MMC specification (**Figure 2D**) (Huang et al., 2022). In *Arabidopsis*, PIN1 is located in the outer layer of the nucellus of the ovule primordia, adjacent to the region formed by MMC. ARF17 and miR160 define the expression domain of PIN1, which contributes to establishment of the local auxin maximum at the ovule apex (**Figure 2D**) (Huang et al., 2022). ARF3 is also involved in ovule development, and its expression is regulated by *trans*-acting small interfering RNAs (known as tasiR-ARFs) (Su et al., 2017). *ARF3* is detected in the central chalazal region, while ARF3m (tasiR-ARF-resistant version) spreads from the chalazal region to the distal nucellus except for the MMC, including the cells adjacent to the MMC, resulting in multiple MMC formation (Su et al., 2017). The expression of *ARF3* in ta-siRNA biogenesis-related mutants (such as *tex1* and *tas3*) extends from the chalazal region to the distal nucellus, which is similar to the expression pattern of ARF3m, indicating that *TEX1* and *TAS3* mediate the expression of ARF3, which restricts the formation of supernumerary MMC formation through non-cell autonomous pathway (**Figure 2C**) (Su et al., 2017).

Similar to auxin, brassinosteroid (BR) also has a gradient in the plant ovule. BR biosynthetic and signaling components genes were expressed exclusively in the sporophytic tissues of the ovule primordia, but not in the MMC (Cai et al., 2022a). Furthermore, BR-deficient mutants and BR-insensitive mutants *bri1-116* and BRASSINOZOLE RESISTANT 1 (BZR1) family quintuple mutant *qui-1* (*bes1-1 bzr1-1 beh1-1 beh3-1beh4-1* quintuple mutant) produced excessive MMCs and further entry into meiosis. These results suggest that BR signaling influences the acquisition of female germline identity (**Figure 2C**) (Cai et al., 2022a). A further study found that BR signaling through the BZR1 transcription factor family and their targeted gene *WRKY23* regulates female germline identity of the subepidermal cells adjacent to the MMC (**Figure 2C**) (Cai et al., 2022a). Furthermore, EPFL-ERf ligand–receptor pairs act upstream of the BZR1 family and coordinate regulation of female germline specification by directly activating the expression of encoded a nucleolar GTP-binding protein, NUCLEOSTEMIN-LIKE 1 (NSN1), which expressed in hypodermal cells surrounding the MMC and restricts these cells, acquiring female germline identity in the ovule primordia (**Figure 2C**) (Cai et al., 2023a). A recent study found that two key epigenetic factors SWR1 and SDG2 cooperate with the ER signaling pathway and their downstream BZR1 transcription factors to regulate female germline development by activating small RNA processor factors *SERRATE* (*SE*), *HYPONASTIC LEAVES 1* (*HYL1*) and *DICER-LIKE 1* (*DCL1*) (**Figure 2D**) (Cai et al., 2023a).

Gibberellins (GAs) are essential for many processes of plant growth and development, such as seed germination, elongation growth, flowering time, and embryo sac development (Achard and Genschik, 2009). The binding of GAs to the gibberellin receptor GIBBERELLIN-INSENSITIVE DWARF1 (GID1) leads to a conformational change in the N-terminal extension of GID1 and inhibits GA action (Miyako et al., 2005, 2007). The DELLA proteins belong to the plant-specific GRAS family and act as GA-signaling repressors, and the formation of GA-GID1-DELLA complex results in the rapid degradation of DELLA proteins, releasing the action of GA by destabilizing and degrading DELLA proteins (Sun, 2010; 2011). The two important GA components—GID1 and DELLA—play important roles in controlling ovule initiation and ovule number, respectively (Ferreira et al., 2017; Maria et al., 2018). In *Arabidopsis*, three genes—*GID1a*, *GID1b*, and *GID1c*—were identified in these plants and considered orthologous to the rice *GID1*. *GID1a* and *GID1b* were specifically expressed in the inner and outer integuments in the ovule, but *GID1c* was not detected in the ovule (Ferreira et al., 2017). Moreover, the *gid1a* single mutants and the *gid1a gid1b* and *gid1a gid1c* double mutants showed compromised fertility mainly caused by maternal defects (Carolina et al., 2014; Maria et al., 2018). The *gid1a gid1b gid1c* triple mutant is complete infertility due to defects in female gametophytes, while MMC development is normal (Carolina et al., 2014). In addition, overexpression of *GID1a* in the nucellus via the ovule-specific *SEEDSTICK* (*STK*) promoter and *CaMV35S* promoter leads to the formation of ovules with multiple MMC-like cells, but only one cell expressed *pKNU::nlsYFP* signal, completed the meiotic division, and entered the gametogenesis process (**Figure 2C**) (Ferreira et al., 2017). A recent study using a GA sensor (GA HACR, hormone-activated Cas9-based repressor) that relies on the GA-sensitive RGA has shown that endogenous bioactive GA hormone accumulates in MMCs and surrounding tissues, which indicates that GAs play roles in ovule primordia development (Dolores et al., 2020). However, the GA-mediated molecular mechanisms in MMC formation are still unclear.

Cytokinins have been hypothesized to play roles in plant cell division and differentiation (Inoue et al., 2001). In *Arabidopsis*, there are six cytokinin receptors: ARABIDOPSIS HISTIDINE KINASES 2 (AHK2), AHK3, AHK4/CRE1/WOL, AtHK1, CKI1, and CKI2/AHK5 (Inoue et al., 2001; Higuchi et al., 2004). Among them, CRE1, AHK2, and AHK3 have high homology within the assumed cytokinin-binding extracellular domain and are abundantly expressed during ovule development (Higuchi et al., 2004; Bencivenga et al., 2012). The *ahk2 3 4* triple mutant ovules developed as finger-like structures and showed defects in the formation of the female gametophyte (Bencivenga et al., 2012). The isopentenyltransferase (IPT) family mediates the biosynthesis of CKs in different organisms, which are expressed during all phases of ovule development in *Arabidopsis*. Furthermore, *AtIPT9* mutants also showed extra MMC-like cells, but the identity of these cells remains to be determined. These results suggest that the IPT family may play roles in the proper differentiation of a single MMC during ovule development (**Figure 2C**) (Ferreira et al., 2023).

## Conclusion and future issues

The development of female gametophytes is a complex process, including MMC specification, FM formation, and its three rounds of continuous mitosis (**Figure 1**) (Chevalier et al., 2011). Along the distal–proximal axis of the *Arabidopsis* ovule primordia, the early ovule primordia can be divided into three regions: nucellus, chalaza, and funiculus (Schneitz et al., 1995). The most distal of these regions is the nucellus, which gives rise to produce female germline (Hou et al., 2021). MMC is the first female germline cell of most flowering plants, distinguished from its surrounding cells by the deposition of β-1,3-glucan (callus) in the cell wall, as well as an enlarged central nucleus, unique histone markers, and specific gene expression profiles (Webb and Gunning, 1990; Schmidt et al., 2011; She et al., 2013). Therefore, the intercellular communication between the female germline and its adjoining cells plays an important role in cell differentiation and reproduction. Given that the MMCs are hard to collect, there remain many questions about MMC formation. In recent times, more advanced technologies have provided opportunities to explore the mechanism of female germline formation (Pinto et al., 2019; Inderdeep et al., 2024). Single-cell transcriptome analysis provides an effective way to investigate complex cellular systems at the single-cell level, reflecting the biological complexity of different cell types and individual tissues. The MMC identity markers *KNUCKLES* (*KNU*) and ARGONAUTE9 (AGO9) are used to determine cell identity and collect candidate genes (Tucker et al., 2012). A recent study found that the β-1,3-glucan metabolism- and plasmodesmata-related genes are required for female gametogenesis in *Arabidopsis* through single-cell transcriptome analysis (Pinto et al., 2024). The concentrated expression of β-1,3-glucanase in the female germline temporarily disrupts the deposition of β-1,3-glucan, which promotes the intercellular communication between adjoining female germline and somatic cells and affects the germline gene expression and histone marks, eventually ultimately resulting in termination of female germline development (Pinto et al., 2024). In the near future, single-cell multiomics technologies in plants will make it possible for us to unravel the differences in genome, epigenome, transcriptome, translatome, proteome, and/or metabolome between the female germline and its adjoining somatic cells.

The formation of the female germline is a complex network, and different pathways are connected to ensure a single MMC formation and its subsequent development. SPL/NZZ seems to be the central regulator in female formation. *WUS* acts downstream of *SPL/NZZ* in MMC development, but the percentage of the *spl/nzz* mutant unable to produce a germline is significantly higher than that in the *wus* mutant, suggesting that SPL/NZZ plays additional functions independently of WUS (**Figure 2B**) (Yang et al., 1999; Gross-Hardt et al., 2002; Lieber et al., 2011). The interaction between SPL/NZZ and phytohormones may create the microenvironment for establishing a female germline. For example, auxin signaling is required for MMC formation in an ARF17- and SPL/NZZ-dependent manner; *ARF17* and *miR160* define the expression domain of PIN1, which contributes to the establishment of the local auxin maximum at the ovule apex and provide spatially restricted information for the proper specification of a single MMC per ovule, suggesting that phytohormones appear to provide the position cue in MMC specification (**Figure 2D**) (Huang et al., 2022). In addition, the expression of *SPL/NZZ* is repressed by the RNA-directed DNA methylation (RdDM) pathway, which is required for restriction of the female germline to a single nucellus cell (**Figure 2C**) (Marta et al., 2020). Future studies may focus on understanding the mechanisms of how and when somatic cells perceive and respond to germline-inducing signals, as well as the mechanisms that inhibit multiple germline cells undergoing meiosis. Further details need to be elucidated on different pathways that lead to regulating germline specification. In addition, external environmental conditions are crucial for plant development, but there have been no reports on the effects of adverse conditions on female germline development. Advanced technology may facilitate the finding of new germline specification factors and their functional mechanisms.

## References

[B1] Achard P. Genschik P. (2009). Releasing the brakes of plant growth: how GAs shutdown DELLA proteins. J. Exp. Bot. 60, 1085–1092. doi: 10.1093/jxb/ern301 19043067

[B2] Bencivenga S. Simonini S. Benkova E. Colombo L. (2012). The transcription factors BEL1 and SPL are required for cytokinin and auxin signaling during ovule development in Arabidopsis. Plant Cell 24, 2886–2897. doi: 10.1105/tpc.112.100164 22786869 PMC3426121

[B3] Berr A. McCallum E. Ménard R. Meyer D. Fuchs J. Dong A.. (2010). Arabidopsis SET DOMAIN GROUP2 is required for H3K4 trimethylation and is crucial for both sporophyte and gametophyte development. Plant Cell 22, 3232–3248. doi: 10.1105/tpc.110.079962 21037105 PMC2990135

[B4] Cai H. Huang Y. Liu L. Zhang M. Chai M. Xi X.. (2023a). Signaling by the EPFL-ERECTA family coordinates female germline specification through the BZR1 family in Arabidopsis. Plant Cell. 35, 1455–1473. doi: 10.1093/plcell/koad032 PMC10118260 36748257

[B5] Cai H. Liu L. Huang Y. Zhu W. Qi J. Xi X.. (2022a). Brassinosteroid signaling regulates female germline specification in Arabidopsis. Curr. Biol. 32, 1102–1114.e1105. doi: 10.1016/j.cub.2022.01.022 35108524

[B6] Cai H. Liu L. Ma S. Aslam M. Qin Y. (2023b). Insights into the role of phytohormones in plant female germline cell specification. Curr. Opin. Plant Biol. 75, 102439. doi: 10.1016/j.pbi.2023.102439 37604069

[B7] Cai H. Ma S. Su H. Liu K. Aslam M. Qin Y. (2022b). Positional signals establishment in the regulation of female germline specification. Seed Biol. 1, 1–7. doi: 10.48130/SeedBio-2022-0006

[B8] Cao L. Wang S. Venglat P. Zhao L. H. Cheng Y. Yee S. J.. (2018). Arabidopsis ICK/KRP cyclin-dependent kinase inhibitors function to ensure the formation of one megaspore mother cell and one functional megaspore per ovule. PloS Genet. 14, e1007230. doi: 10.1371/journal.pgen.1007230 29513662 PMC5858843

[B9] Carolina G. G. Hu J. Urbez C. Gomez M. D. Taiping S. Perez-Amador M. A. (2014). Role of the gibberellin receptors GID1 during fruit-set in Arabidopsis. Plant J. 79, 1020–1032. doi: 10.1111/tpj.12603 PMC4403254 24961590

[B10] Chen G. Sun J. Liu M. Liu J. Yang W. (2014). SPOROCYTELESS is a novel embryophyte-specific transcription repressor that interacts with TPL and TCP proteins in Arabidopsis. J. Genet. Genomics 41, 617–625. doi: 10.1016/j.jgg.2014.08.009 25527103

[B11] Chevalier É. Loubert-Hudon A. Zimmerman E. Matton D. (2011). Cell-cell communication and signalling pathways within the ovule: from its inception to fertilization. New Phytol. 192, 13–28. doi: 10.1111/j.1469-8137.2011.03836.x 21793830

[B12] Da Ines O. Abe K. Goubely C. Gallego M. White C. (2012). Differing requirements for RAD51 and DMC1 in meiotic pairing of centromeres and chromosome arms in Arabidopsis thaliana. PloS Genet. 8, e1002636. doi: 10.1371/journal.pgen.1002636 22532804 PMC3330102

[B13] Deborah W. Loren P. Hester M W. Lois M. Philip W T. Elizabeth M. (2005). Nomenclature of the ARID family of DNA-binding proteins. Genomics 86, 242–251. doi: 10.1016/j.ygeno.2005.03.013 15922553

[B14] Desvoyes B. Gutierrez C. (2020). Roles of plant retinoblastoma protein: cell cycle and beyond. EMBO J. 39, e105802. doi: 10.15252/embj.2020105802 32865261 PMC7527812

[B15] De Veylder L. Beeckman T. Beemster G. T. S. Krols L. Terras F. Landrieu I.. (2001). Functional analysis of cyclin-dependent kinase inhibitors of arabidopsis. Plant Cell 13, 1653–1668. doi: 10.1105/TPC.010087% 11449057 PMC139548

[B16] Dolores G. M. Daniela B. T. Clara F. A. Pablo T. Alonso J. M. Perez-Amador M. A. (2020). Gibberellin-mediated RGA-LIKE1 degradation regulates embryo sac development in Arabidopsis. J. Exp. Bot. 71, 7059–7072. doi: 10.1093/jxb/eraa395 32845309 PMC7906783

[B17] Ferreira L. G. de Alencar Dusi D. M. Irsigler A. S. T. Gomes A. C. M. M. Mendes M. A. Colombo L.. (2017). GID1 expression is associated with ovule development of sexual and apomictic plants. Plant Cell Rep. 37, 293–306. doi: 10.1007/s00299-017-2230-0 29080908

[B18] Ferreira L. Dusi D. Irsigler A. Gomes A. Florentino L. Mendes M.. (2023). Identification of IPT9 in Brachiaria brizantha (syn. Urochloa brizantha) and expression analyses during ovule development in sexual and apomictic plants. Mol. Biol. Rep. 50, 4887–4897. doi: 10.1007/s11033-023-08295-7 37072653 PMC10209240

[B19] Gross-Hardt R. Lenhard M. Laux T. (2002). WUSCHEL signaling functions in interregional communication during Arabidopsis ovule development. Genes Dev. 16, 1129–1138. doi: 10.1101/gad.225202 12000795 PMC186242

[B20] Havecker E. Wallbridge L. Hardcastle T. Bush M. Kelly K. Dunn R.. (2010). The Arabidopsis RNA-directed DNA methylation argonautes functionally diverge based on their expression and interaction with target loci. Plant Cell 22, 321–334. doi: 10.1105/tpc.109.072199 20173091 PMC2845420

[B21] Hernandez-Lagana E. Autran D. (2020). H3.1 eviction marks female germline precursors in arabidopsis. Plants (Basel) 9, 1322. doi: 10.3390/plants9101322 33036297 PMC7600056

[B22] Hernandez-Lagana E. Rodriguez-Leal D. Lua J. Vielle-Calzada J. P. (2016). A multigenic network of ARGONAUTE4 clade members controls early megaspore formation in arabidopsis. Genetics 204, 1045–1056. doi: 10.1534/genetics.116.188151 27591749 PMC5105840

[B23] Higuchi M. Pischke M. S. Mähönen A. P. Miyawaki K. Hashimoto Y. Seki M.. (2004). In planta functions of the Arabidopsis cytokinin receptor family. Proc. Natl. Acad. Sci. U.S.A. 101, 8821–8826. doi: 10.1073/pnas.0402887101 15166290 PMC423279

[B24] Hou Z. Liu Y. Zhang M. Zhao L. Jin X. Liu L.. (2021). High-throughput single-cell transcriptomics reveals the female germline differentiation trajectory in Arabidopsis thaliana. Commun. Biol. 4, 1149. doi: 10.1038/s42003-021-02676-z 34599277 PMC8486858

[B25] Huang J. Zhao L. Malik S. Gentile B. R. Xiong V. Arazi T.. (2022). Specification of female germline by microRNA orchestrated auxin signaling in Arabidopsis. Nat. Commun. 13, 6960. doi: 10.1038/s41467-022-34723-6 36379956 PMC9666636

[B26] Inderdeep K. Renu K. Monika K. (2024). Understanding megasporogenesis through model plants: contemporary evidence and future insights. Int. J. Dev. Biol. 68, 9–17. doi: 10.1387/ijdb.230222mk 38591693

[B27] Inoue T. Higuchi M. Hashimoto Y. Seki M. Kobayashi M. Kato T.. (2001). Identification of CRE1 as a cytokinin receptor from Arabidopsis. Nature 409, 1060–1063. doi: 10.1038/35059117 11234017

[B28] Jacob Y. Bergamin E. Donoghue M. T. Mongeon V. LeBlanc C. Voigt P.. (2014). Selective methylation of histone H3 variant H3.1 regulates heterochromatin replication. Science 343, 1249–1253. doi: 10.1126/science.1248357 24626927 PMC4049228

[B29] Juan Antonio T. A. Larry C F. Hong W. (2011). Analyses of phylogeny, evolution, conserved sequences and genome-wide expression of the ICK/KRP family of plant CDK inhibitors. Ann. Bot. 107, 1141–1157. doi: 10.1093/aob/mcr034 21385782 PMC3091803

[B30] Justyna W. Jian X. Daniela S. Philip B B. Kamil R. Ikram B.. (2006). Polar PIN localization directs auxin flow in plants. Science 312, 883. doi: 10.1126/science.1121356 16601151

[B31] Li L. Wu W. Zhao Y. Zheng B. (2017). A reciprocal inhibition between ARID1 and MET1 in male and female gametes in Arabidopsis. J. Integr. Plant Biol. 59, 657–668. doi: 10.1111/jipb.12573 28782297

[B32] Lieber D. Lora J. Schrempp S. Lenhard M. Laux T. (2011). Arabidopsis WIH1 and WIH2 genes act in the transition from somatic to reproductive cell fate. Curr. Biol. 21, 1009–1017. doi: 10.1016/j.cub.2011.05.015 21658947

[B33] Liu W. Gallego-Bartolomé J. Zhou Y. Zhong Z. Wang M. Wongpalee S. P.. (2021). Ectopic targeting of CG DNA methylation in Arabidopsis with the bacterial SssI methyltransferase. Nat. Communication 12, 3130. doi: 10.1038/s41467-021-23346-y PMC8149686 34035251

[B34] Lorena A. ,. S. Caroline M. Benjamin S. Juan Manuel V. Silvina C P. Mathieu I.. (2023). TRIMETHYLGUANOSINE SYNTHASE1 mutations decanalize female germline development in Arabidopsis. New Phytol. 240, 597–612. doi: 10.1111/nph.19179 37548040

[B35] Lv Y. Li J. Wang Z. Liu Y. Jiang Y. Li Y.. (2024). Polycomb proteins RING1A/B promote H2A monoubiquitination to regulate female gametophyte development in Arabidopsis. J. Exp. Bot. 75, 4822–4836. doi: 10.1093/jxb/erae208 38717070

[B36] Maria D. ,. G. Daniela B.-T. Ernesto E. Maite S.-S. Ines S. Asier B.-M.. (2018). Gibberellins negatively modulate ovule number in plants. Development 145, dev163865. doi: 10.1242/dev.163865 29914969 PMC6053663

[B37] Marston A. L. (2014). Chromosome segregation in budding yeast: sister chromatid cohesion and related mechanisms. Genetics 196, 31–63. doi: 10.1534/genetics.112.145144 24395824 PMC3872193

[B38] Marta A. ,. M. Rosanna P. Mara C. Edoardo V. Stefano G. Sara C P.. (2020). The RNA-dependent DNA methylation pathway is required to restrict SPOROCYTELESS/NOZZLE expression to specify a single female germ cell precursor in Arabidopsis. Development 147, dev194274. doi: 10.1242/dev.194274 33158925 PMC7758631

[B39] Matias-Hernandez L. Battaglia R. Galbiati F. Rubes M. Eichenberger C. Grossniklaus U.. (2010). VERDANDI is a direct target of the MADS domain ovule identity complex and affects embryo sac differentiation in Arabidopsis. Plant Cell 22, 1702–1715. doi: 10.1105/tpc.109.068627 20581305 PMC2910977

[B40] Mayer K. F. Schoof H. Haecker A. Lenhard M. Jurgens G. Laux T. (1998). Role of WUSCHEL in regulating stem cell fate in the Arabidopsis shoot meristem. Cell 95, 805–815. doi: 10.1016/s0092-8674(00)81703-1 9865698

[B41] Miyako U.-T. Masatoshi N. Ashikari M. Makoto M. (2007). Gibberellin receptor and its role in gibberellin signaling in plants. Annu. Rev. Plant Biol. 58, 183–198. doi: 10.1146/annurev.arplant.58.032806.103830 17472566

[B42] Miyako U.-T. Motoyuki A. Masatoshi N. Hironori I. Etsuko K. Masatomo K.. (2005). GIBBERELLIN INSENSITIVE DWARF1 encodes a soluble receptor for gibberellin. Nature 437, 693–698. doi: 10.1038/nature04028 16193045

[B43] Nonomura K. I. Miyoshi K. Eiguchi M. Suzuki T. Miyao A. Hirochika H.. (2003). The MSP1 gene is necessary to restrict the number of cells entering into male and female sporogenesis and to initiate anther wall formation in rice. Plant Cell 15, 1728–1739. doi: 10.1105/tpc.012401 12897248 PMC167165

[B44] Okada T. Endo M. Singh M. B. Bhalla P. L. (2005). Analysis of the histone H3 gene family in Arabidopsis and identification of the male-gamete-specific variant AtMGH3. Plant J. 44, 557–568. doi: 10.1111/j.1365-313X.2005.02554.x 16262706

[B45] Olmedo-Monfil V. Duran-Figueroa N. Arteaga-Vazquez M. Demesa-Arevalo E. Autran D. Grimanelli D.. (2010). Control of female gamete formation by a small RNA pathway in Arabidopsis. Nature 464, 628–U200. doi: 10.1038/nature08828 20208518 PMC4613780

[B46] Pagnussat G. Alandete-Saez M. Bowman J. Sundaresan V. (2009). Auxin-dependent patterning and gamete specification in the Arabidopsis female gametophyte. Science 324, 1684–1689. doi: 10.1126/science.1167324 19498110

[B47] Pauline E. J. Assaf M. Mathieu I. Tadashi S. Nir O. Frédéric B. (2008). Retinoblastoma and its binding partner MSI1 control imprinting in Arabidopsis. PloS Biol. 6, e194. doi: 10.1371/journal.pbio.0060194 18700816 PMC2504488

[B48] Pinto S. C. Leong W. H. Tan H. McKee L. Prevost A. Ma C.. (2024). Germline β-1,3-glucan deposits are required for female gametogenesis in Arabidopsis thaliana. Nat. Commun. 15, 5875. doi: 10.1038/s41467-024-50143-0 38997266 PMC11245613

[B49] Pinto S. C. Mendes M. A. Coimbra S. Tucker M. R. (2019). Revisiting the female germline and its expanding toolbox. Trends Plant Sci. 24, 455–467. doi: 10.1016/j.tplants.2019.02.003 30850278

[B50] Qin Y. Zhao L. H. Skaggs M. I. Andreuzza S. Tsukamoto T. Panoli A.. (2014). ACTIN-RELATED PROTEIN6 regulates female meiosis by modulating meiotic gene expression in arabidopsis. Plant Cell 26, 1612–1628. doi: 10.1105/tpc.113.120576 24737671 PMC4036575

[B51] Rodriguez-Leal D. Leon-Martinez G. Abad-Vivero U. Vielle-Calzada J. P. (2015). Natural variation in epigenetic pathways affects the specification of female gamete precursors in arabidopsis. Plant Cell 27, 1034–1045. doi: 10.1105/tpc.114.133009 25829442 PMC4558685

[B52] Rosa M. Von M. H. Cigliano R. Schlögelhofer P. Mittelsten O. S. (2013). The Arabidopsis SWR1 chromatin-remodeling complex is important for DNA repair, somatic recombination, and meiosis. Plant Cell 25, 1990–2001. doi: 10.1105/tpc.112.104067 23780875 PMC3723608

[B53] Schiefthaler U. Balasubramanian S. Sieber P. Chevalier D. Wisman E. Schneitz K. (1999). Molecular analysis of NOZZLE, a gene involved in pattern formation and early sporogenesis during sex organ development in Arabidopsis thaliana. Proc. Natl. Acad. Sci. U.S.A. 96, 11664–11669. doi: 10.1073/pnas.96.20.11664 10500234 PMC18091

[B54] Schmidt A. Wuest S. E. Vijverberg K. Baroux C. Kleen D. Grossniklaus U. (2011). Transcriptome analysis of the arabidopsis megaspore mother cell uncovers the importance of RNA helicases for plant germline development. PloS Biol. 9, 1–19. doi: 10.1371/journal.pbio.1001155 PMC3176755 21949639

[B55] Schneitz K. Hülskamp M. Pruitt R. E. (1995). Wild-type ovule development in Arabidopsis thaliana: A light microscope study of cleared whole-mount tissue. Plant J. 7, 731–749. doi: 10.1046/j.1365-313X.1995.07050731.x

[B56] She W. Grimanelli D. Rutowicz K. Whitehead M. W. Puzio M. Kotlinski M.. (2013). Chromatin reprogramming during the somatic-to-reproductive cell fate transition in plants. Development 140, 4008–4019. doi: 10.1242/dev.095034 24004947

[B57] Sheridan W. F. Avalkina N. A. Shamrov I. I. Batygina T. B. Golubovskaya I. N. (1996). The mac1 gene: controlling the commitment to the meiotic pathway in maize. Genetics 142, 1009–1020. doi: 10.1093/genetics/142.3.1009 8849906 PMC1207000

[B58] Shirley P. Doron G. (2008). E2F - at the crossroads of life and death. Trends Cell Biol. 18, 528–535. doi: 10.1016/j.tcb.2008.08.003 18805009

[B59] Singh M. Goel S. Meeley R. B. Dantec C. Parrinello H. Michaud C.. (2011). Production of viable gametes without meiosis in maize deficient for an ARGONAUTE protein. Plant Cell 23, 443–458. doi: 10.1105/tpc.110.079020 21325139 PMC3077773

[B60] Su Z. X. Zhao L. H. Zhao Y. Y. Li S. F. Won S. Cai H. Y.. (2017). The THO complex non-cell-autonomously represses female germline specification through the TAS3-ARF3 module. Curr. Biol. 27, 1597–1609. doi: 10.1016/j.cub.2017.05.021 28552357 PMC5544534

[B61] Sun T. (2010). Gibberellin-GID1-DELLA: a pivotal regulatory module for plant growth and development. Plant Physiol. 154, 567–570. doi: 10.1104/pp.110.161554 PMC2949019 20921186

[B62] Sun T. (2011). The molecular mechanism and evolution of the GA-GID1-DELLA signaling module in plants. Curr. Biol. 21, R338–R345. doi: 10.1016/j.cub.2011.02.036 21549956

[B63] Tovar-Aguilar A. Grimanelli D. Acosta-García G. Vielle-Calzada J. Badillo-Corona J. Durán-Figueroa N. (2024). The miRNA822 loaded by ARGONAUTE9 modulates the monosporic female gametogenesis in Arabidopsis thaliana. Plant Reprod. 37, 243–258. doi: 10.1007/s00497-023-00487-2 38019279

[B64] Tucker M. R. Okada T. Hu Y. Scholefield A. Taylor J. M. Koltunow A. M. (2012). Somatic small RNA pathways promote the mitotic events of megagametogenesis during female reproductive development in Arabidopsis. Development 139, 1399–1404. doi: 10.1242/dev.075390 22399683

[B65] Wang H. Fowke L. C. Crosby W. L. (1997). A plant cyclin-dependent kinase inhibitor gene. Nature 386, 451–452. doi: 10.1038/386451a0 9087400

[B66] Wang C. J. Nan G.-L. Kelliher T. Timofejeva L. Vernoud V. Golubovskaya I. N.. (2012). Maize multiple archesporial cells 1 (mac1), an ortholog of rice TDL1A, modulates cell proliferation and identity in early anther development. Development 139, 2594–2603. doi: 10.1242/dev.077891 22696296 PMC4496874

[B67] Webb M. C. Gunning B. E. S. (1990). Embryo sac development in Arabidopsis thaliana. Sexual Plant Reprod. 3, 244–256. doi: 10.1007/BF00202882

[B68] Wei B. Zhang J. Pang C. Yu H. Guo D. Jiang H.. (2015). The molecular mechanism of SPOROCYTELESS/NOZZLE in controlling Arabidopsis ovule development. Cell Res. 25, 121–134. doi: 10.1038/cr.2014.145 25378179 PMC4650584

[B69] Wu W. Alami S. Luk E. Wu C. Sen S. Mizuguchi G.. (2005). Swc2 is a widely conserved H2AZ-binding module essential for ATP-dependent histone exchange. Nat. Struct. Mol. Biol. 12, 1064–1071. doi: 10.1038/nsmb1023 16299513

[B70] Yan D. Duermeyer L. Leoveanu C. Nambara E. (2014). The functions of the endosperm during seed germination. Plant Cell Physiol. 55, 1521–1533. doi: 10.1093/pcp/pcu089 24964910

[B71] Yang W. Sundaresan V. (2000). Genetics of gametophyte biogenesis in Arabidopsis. Curr. Opin. Plant Biol. 3, 53–57. doi: 10.1016/s1369-5266(99)00037-0 10679449

[B72] Yang W. C. Ye D. Jian X. Sundaresan V. (1999). The SPOROCYTELESS gene of Arabidopsis is required for initiation of sporogenesis and encodes a novel nuclear protein. Genes Dev. 13, 2108–2117. doi: 10.1101/gad.13.16.2108 10465788 PMC316961

[B73] Yao X. Z. Yang H. D. Zhu Y. X. Xue J. S. Wang T. H. Song T.. (2018). The canonical E2Fs are required for germline development in arabidopsis. Front. Plant Sci. 9. doi: 10.3389/fpls.2018.00638 PMC5962754 29868091

[B74] Yin X. Romero-Campero F. Reyes P. Yan P. Yang J. Tian G.. (2021). H2AK121ub in Arabidopsis associates with a less accessible chromatin state at transcriptional regulation hotspots. Nat. Commun. 12, 315. doi: 10.1038/s41467-020-20614-1 33436613 PMC7804394

[B75] Yoshikawa M. Peragine A. Park M. Poethig R. (2005). A pathway for the biogenesis of trans-acting siRNAs in Arabidopsis. Genes Dev. 19, 2164–2175. doi: 10.1101/gad.1352605 16131612 PMC1221887

[B76] Zhao X. A. Bramsiepe J. Van Durme M. Komaki S. Prusicki M. A. Maruyama D.. (2017). RETINOBLASTOMA RELATED1 mediates germline entry in Arabidopsis. Science 356, eaaf6532. doi: 10.1126/science.aaf6532 28450583

[B77] Zhao L. H. Cai H. Y. Su Z. X. Wang L. L. Huang X. Y. Zhang M.. (2018). KLU suppresses megasporocyte cell fate through SWR1-mediated activation of WRKY28 expression in Arabidopsis. Proc. Natl. Acad. Sci. United States America 115, E526–E535. doi: 10.1073/pnas.1716054115 PMC5776990 29288215

[B78] Zhao X. Palma J. D. Oane R. Gamuyao R. Luo M. Chaudhury A.. (2008). OsTDL1A binds to the LRR domain of rice receptor kinase MSP1, and is required to limit sporocyte numbers. Plant J. 54, 375–387. doi: 10.1111/j.1365-313X.2008.03426.x 18248596 PMC2408674

[B79] Zheng B. He H. Zheng Y. Wu W. Sheila M. (2014). An ARID domain-containing protein within nuclear bodies is required for sperm cell formation in Arabidopsis thaliana. PloS Genet. 10, e1004421. doi: 10.1371/journal.pgen.1004421 25057814 PMC4109846

[B80] Zhou Y. Li G. Brandizzi F. Fowke L. Wang H. (2003). The plant cyclin-dependent kinase inhibitor ICK1 has distinct functional domains for *in vivo* kinase inhibition, protein instability and nuclear localization. Plant J. 35, 476–489. doi: 10.1046/j.1365-313x.2003.01821.x 12904210

